# A novel method for genome-wide profiling of dynamic host-pathogen interactions using 3′ end enriched RNA-seq

**DOI:** 10.1038/s41598-017-08700-9

**Published:** 2017-08-17

**Authors:** Jie Li, Liangliang He, Yun Zhang, Chunyi Xue, Yongchang Cao

**Affiliations:** 1 State Key Laboratory of Biocontrol, Guangdong Province Key Laboratory of Pharmaceutical Functional Genes, College of Life Sciences, Sun Yat-sen University, Guangzhou, People’s Republic of China; 2 Guangdong Wen’s Foodstuffs Group Co., Ltd. Yunfu, Guangdong, China

## Abstract

Marek’s disease is a contagious lymphoproliferative disease of chickens and typical model of viral oncogenesis. Mapping changes or different states over the course of infection for both host and pathogen would provide important insights into dynamic host-pathogen interactions. Here we introduced 3′ end enriched RNA-seq as a novel method to study host-pathogen interactions in chicken embryo fibroblasts cells challenged with Marek’s disease virus. The method allowed accurate profiling of gene expression and alternative polyadenylation sites for host and pathogen simultaneously. We totally identified 476 differentially expressed genes and 437 APA switching genes in host, including switching in tandem 3′ UTRs and switching between coding region and 3′ UTR. Most of these genes were related to innate immunity, apoptosis and metabolism, but two sets of genes overlapped a little, suggesting two complementary mechanisms in gene regulation during MDV infection. In summary, our results provided a relatively comprehensive insight into dynamic host-pathogen interactions in regulation of gene transcription during infection of Marek’s disease virus and suggested that 3′ end enriched RNA-seq was a promising method to investigate global host-pathogen interactions.

## Introduction

Virus infection induces and suppresses host gene expression on a global level. Microarray-based and next generation sequencing-based transcriptomic approaches have been used to study host-pathogen interactions for many years. However, our knowledge of the response of host to virus infection is limited, and host-pathogen interactions were often only investigated from the host level. Viruses are obligatory intracellular parasites that require the host machinery to replicate. In theory, these infected cells contain transcribed RNAs from both host and virus, although virus RNAs account only a tiny proportion. Mapping changes or states over the course of infection for both host and pathogen would have the potential to generate important insights into dynamic host-pathogen interactions.

Alternative polyadenylation (APA) is a common regulatory mechanism of gene expression that generates messenger RNAs (mRNAs) with distinct 3′ untranslated regions (3′ UTRs) or coding sequences of different isoforms. With the development of 3′ end sequencing technology, it has been shown that more than half of genes exhibit alternative polyadenylation in human, mouse and other model species^1, 2^. The resulting transcripts may not only exhibit an altered coding potential, but also harbor a distinct set of cis-regulatory elements for microRNAs and other long non-coding RNAs as well as RNA-binding proteins which may significantly change mRNA stability, localization and translation^3–6^. APA consequently increases the complexity of eukaryotic transcriptomes and polyadenylation sites located within introns can result in conversion of an internal exon to a 3′ terminal exon or in usage of a 3′ terminal exon that is otherwise skipped. APA was reported to take part in T-cell activation, neuronal activity, development and several diseases^7–10^. Therefore, it is suggested that APA may also play an important role in host pathogen interactions. However there are not much related studies. Besides, by targeted sequencing ~100 bp of 3′ UTR end from polyA tail, 3′ end enriched RNA-seq could reduce the gap between the RNA quantity of the host and the virus, thus make it possible to obtain information from host and virus together.

Marek’s disease virus (MDV) is a DNA virus with a large genome size of ~176 kb and causes a contagious lymphoproliferative disorder of chickens. To control the disease, live attenuated and non-oncogenic vaccines were used worldwide in the poultry industry^11, 12^. However, administration of vaccination cannot prevent the virus transmission, resulting in the emergence of MDV with enhanced virulence. Therefore, new vaccines and vaccine regimens are needed in the future to control the disease^13^. MDV is also considered as a unique model of viral oncogenesis^14^, but there is still limited knowledge of virus and host interactions during Marek’s disease pathogenesis and oncogenesis^15^.

In this study, we used a novel method to profile differential APA sites switching events in chicken embryo fibroblasts cells challenged with Md5 strains and tried to get more details from the aspects of both host and virus. Our results gave a relatively comprehensive insight into dynamic host-pathogen interactions in gene transcription regulation during Marek’s disease virus infection and suggested that 3′ end enriched RNA-seq may be a promising method to study genome-wide host-pathogen interactions.

## Results

### Poly(A) sites profiling of host in chicken embryo fibroblasts cells

**Figure 1:**
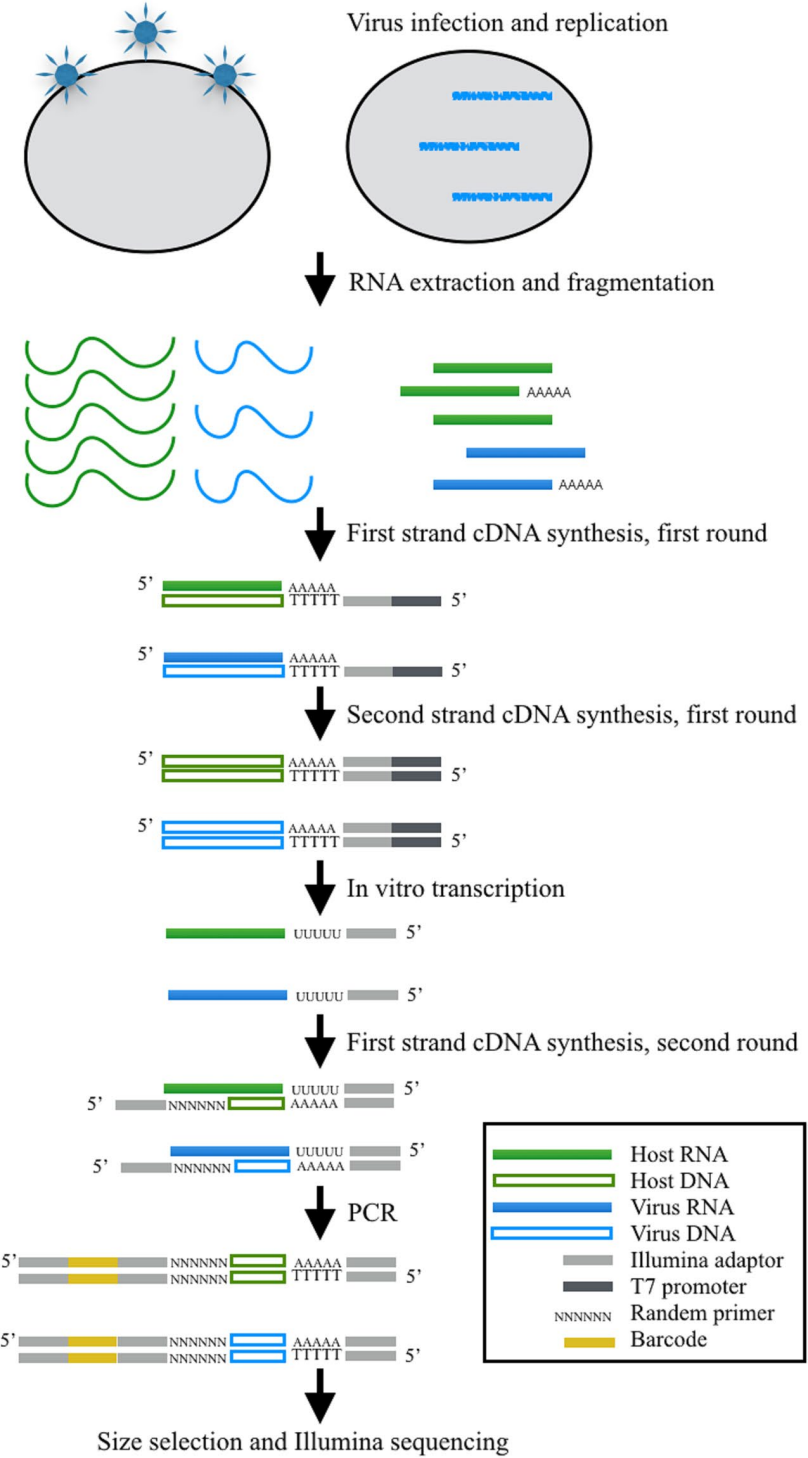
Workflow for 3′ end enriched RNA-seq of host and pathogen together.

To study the dynamic host-pathogen interactions of polyadenylation pattern and expression profile during Marek’s disease virus infection, we used SAPAS method combined with *in vitro* transcription (IVT) and magnetic beads purification^7^ to obtain high-resolution profiling of poly(A) site information and gene expression in embryo fibroblasts cells at five different time points (18 h, 36 h, 54 h, 72 h, 108 h) post-infection. In order to figure out the relationship between virulence of MDV and host-pathogen interactions, we challenged chicken embryo fibroblasts cells with MD5 strain. Samples, including the negative control group, were collected at each time point. Thus, in total ten IVT-SAPAS libraries (U18, C18, U36, C36, U54, C54, U72, C72, U108, C108) were constructed and sequenced (Fig. 1).

**Figure 2:**
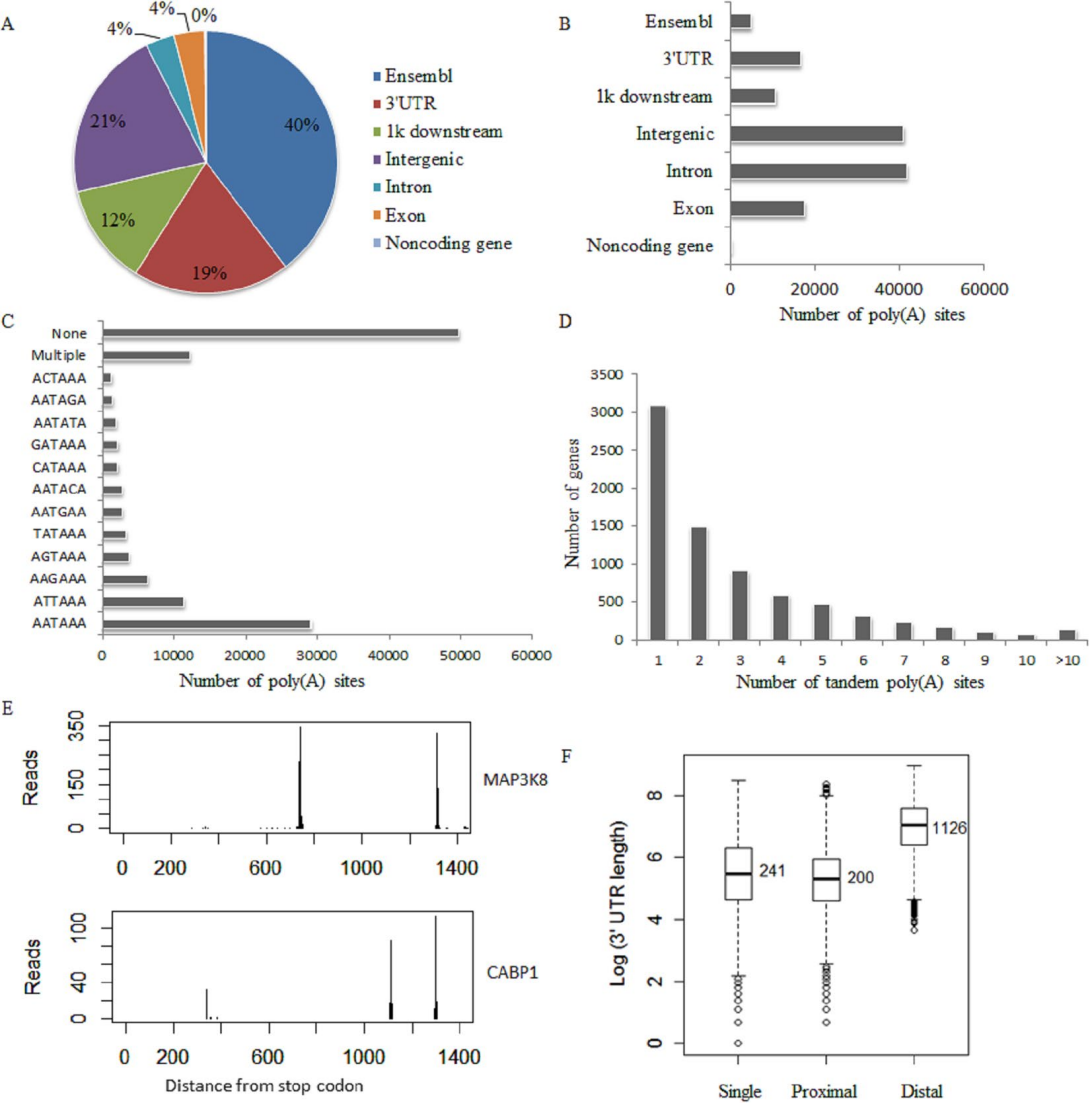
Sequencing reads and poly(A) sites in host genes. (**A**) Genomic locations of sequencing reads. The genomic annotation of host was downloaded from UCSC by selecting Ensembl Genes track and the 3′ UTR tail of these genes were annonated as Ensembl. (**B**) Distribution of poly(A) sites in genome. (**C**) Signal using of poly(A) sites. If none of common hexamers was identified in upstream of poly(A) sites, it was classified into none category. (**D**) Genes with different numbers of tandem poly(A) sites. (**E**) Examples of genes with two and three 3′ UTR isoforms (MAP3K8 and CABP1). (**F**) Histograms of the distances between stop codons and poly(A) sites in genes with single poly(A) sites and distances between stop codons and closest poly(A) sites and longest poly(A) sites in genes with APA in the 3′-end.

A total of 226 million raw reads were generated by Illumina GA sequencing. After mapping to the chicken genome and conducting internal priming filtering, about 85.9 million reads were obtained for further poly(A) site analysis (Table 1). More than 71% reads were located at 3′ UTR region. And due to the limitation of current chicken genome annotation, 21% reads were located at intergenic region (Fig. 2A). These reads were further clustered into 132,461 poly(A) sites at a strict control (at least 10 supporting reads for each site). As little is known about poly(A) sites in chicken, 96% of the 132,461 poly(A) sites were found to be novel. Overall, we found that 16,525, 17,559 and 41,643 poly(A) sites were located in the 3′ UTR, exon and intron region respectively (Fig. 2B). Moreover, 40,909 and 10,761 poly(A) sites were found to locate in the intergenic region and the 1k downstream of 3′ UTR (Fig. 2B). These data suggest an important regulation role of alternative polyadenylation in the chicken genome. Further, the polyadenylation signal hexamer AAUAAA and its close variants were identified in the region 10-40 bp upstream of most poly(A) sites (Fig. 2C) and the frequencies of these hexamers were relatively consistent with the results reported in mammals^2^.

**Table 1:** Summary of SAPAS data from Illumina sequencing.

	U18	C18	U36	C36	U54	C54	U72	C72	U108	C108	Combined
Raw reads	24847601	25522502	27602618	21648980	25288952	23982924	20100806	23064757	13666345	20633362	226358847
Uniquely mapped to virus genome	431	46892	270	83175	876	98766	448	317437	0	341923	890218
Uniquely mapped to host genome	17818426	18132705	20197042	17128230	17042973	17480431	16558407	18590570	10859282	15206581	169014647
Mapped to host nuclear genome	12779656	13007090	15322750	12973011	11918772	11653615	11525383	13148115	8313586	10990038	121632016
After IP filter	8939592	9075591	11030026	9413705	7690603	7526887	8334185	10245822	6094677	7580340	85931428
Genes sampled	11997	12083	12178	12096	11985	12061	11769	12010	11516	11997	11222
Cleavage clusters	127164	137684	116955	94862	143798	131320	90983	90967	76408	107660	132461
Known polyA sites	4420	4498	4620	4484	4443	4371	4392	4486	4206	4410	4881
Novel polyA sites	122744	133186	112335	90378	139355	126949	86591	86481	72202	103250	127580

The poly(A) sites identified above covered 11,222 genes in total. In general, nearly 80% of these genes have at least two poly(A) sites. We then grouped the annotated 3′ UTR into tandem 3′ UTRs by stop codon and combined these isoforms with poly(A) sites profiling as described before^7^. The result showed that 7,008 genes had tandem 3′ UTRs. Among these genes, 59.4% genes had at least two tandem APA sites. On average, 2.9 tandem APA sites in 3′ UTR were detected per gene (Fig. 2D,E). The average distribution of the distances between poly(A) sites and stop codon was about 900 bp and the median value was 621 bp. The median values of distances between the stop codon and the proximal and distal poly(A) sites were 200 bp and 1,126 bp, respectively (Fig. 2F). In summary, these results demonstrate a detailed landscape of poly(A) sites usage in chicken and indicate an inherent vice of gene annotation.

### Profiling of poly(A) sites of Marek’s disease virus

As virus transcribes through host cell’s transcription machinery, virus information were also expected to obtain from generated sequencing reads of infected cells. Traditional RNA sequencing method was limited to the very small quantity of RNAs from virus in comparison with host cells. However, the 3′ end enriched method made it possible for us to quantify the profiling of virus genes accurately as it narrows the gap between virus and host cell through targeted sequencing 3′ end.

**Figure 3:**
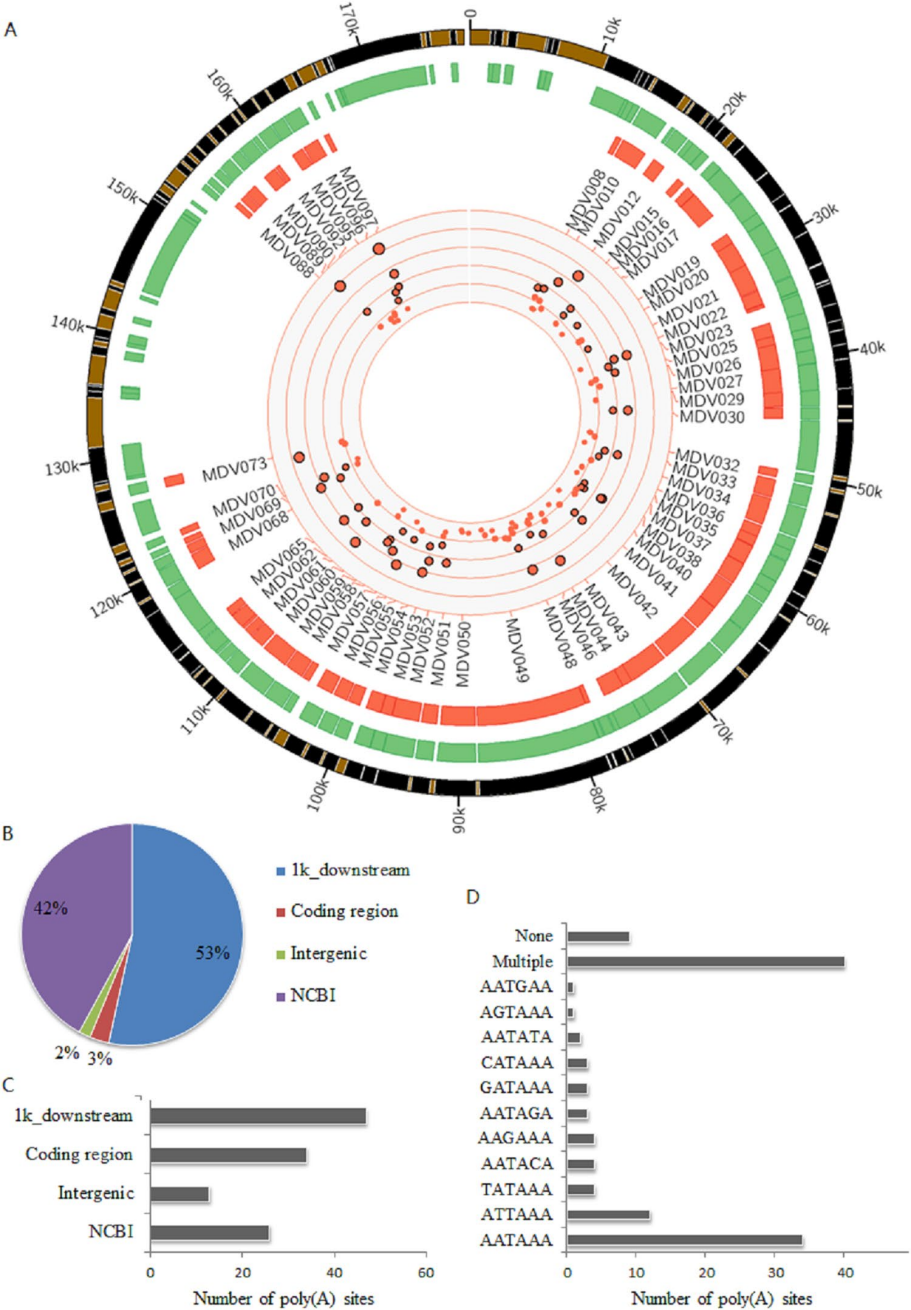
Sequencing reads and poly(A) sites in viral genes. (**A**) Landscape of APA sites profiling for viral genes. The track with green color represents the genome annotation from NCBI. The track with red color represents the expressed genes in our analysis. The red points in the center are the sequenced poly(A) sites in the genome and those with black stroke-color represent that their supporting reads are larger than 100. (**B**) Genomic locations of sequencing reads. The genomic annotation of virus was downloaded from NCBI and the 3′ UTR tail of these genes were annonated as NCBI. (**C**) Distribution of poly(A) sites in genome. (**D**) Signal using of poly(A) sites. If none of common hexamers was identified in upstream of poly(A) sites, it was classified into none category.

After mapping sequencing reads to MDV genome and conducting internal priming filtering as done in host, about 0.9 million reads were obtained for MDV (Table 1, Fig. 3A,B). The proportion of virus reads partly reflected the proliferation or infection status of the virus in host cells. The proportion of MDV increased with time. The MDV reads could be clustered into 120 poly(A) sites covering 60 genes after a strict control. Nearly 30% of the poly(A) sites located in protein coding region and about half of the poly(A) sites were in 3′ UTR or downstream (Fig. 3C). Among these genes, 25 genes had multiple poly(A) sites. The polyadenylation signal hexamer AAUAAA and its close variants were also identified in the region 10–40 bp upstream of the poly(A) sites (over 85%) and AAUAAA, AUUAAA were the two hexamers used most frequently as in host cells (Fig. 3D).

### Dynamic gene expression regulation in host-pathogen interactions

**Figure 4:**
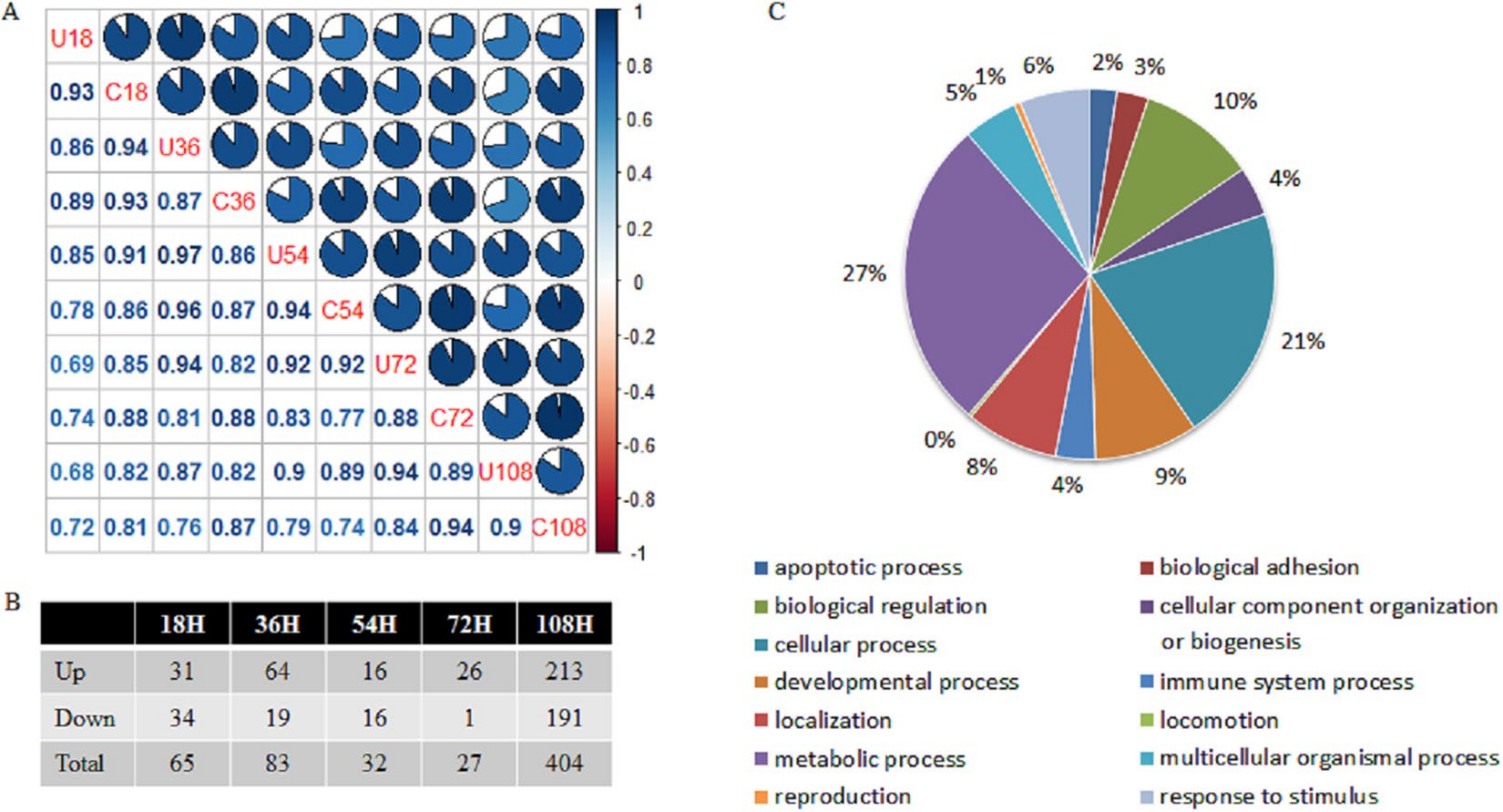
Differentially expressed genes in host-pathogen interactions. (**A**) Pearson correlation coefficients of gene expression profiles among samples. (**B**) Summary of genes with differential expression profiles between samples at different time points. (**C**) Functional classification of differentially expressed genes.

It has been reported that SAPAS and other 3′-end sequencing methods are as accurate as RNA-Seq approaches for digital gene expression^7^. The normalized expression level of each gene with tandem 3′ UTR was measured by reads per million mapped reads (RPM) in the gene. In total, global profiles of gene expression were generally well correlated between samples, with Pearson correlation coefficients ranging from 0.72 to 0.97 (Fig. 4A).

Using the RPM expression data, differences in gene expression between unchallenged and challenged samples at different time points were compared. Genes that changed nearly more than 2-fold and had a false discovery rate (FDR) less than 0.01 were considered to be genes regulated differentially. We found 65, 83, 32, 27 and 404 genes were differentially regulated at 18 h, 36 h, 54 h, 72 h and 108 h, respectively (Fig. 4B). In total, we detected 476 differentially regulated genes between the unchallenged and challenged samples, among which 234 genes were up-regulated, 226 genes were down-regulated and 16 genes were up-regulated and down-regulated at different time points. These genes covered apoptotic process, cellular process, immune system process and metabolic process etc (Fig. 4C). Then we selected 4 up-regulated genes and 4 down-regulated genes to validate gene expression change with qPCR and the qPCR result was consistent with the SAPAS sequencing result (see Supplementary Fig. S1).

At the early stage of MDV infection (18 h), we ranked the fold change of gene expression before and after infection. Top 10 fold changed genes were ISG12, IFI27L2, CMPK2, COL9A2, ENPP2, EX-FABP, LY6E, FMOD, PARP9 and IFIH1. Among them, COL9A2, ENPP2 and FMOD were only significantly up-regulated at 18 h and the other genes were up-regulated at all time points in the challenged samples. According to GeneCards website^16^, the functions of these 10 genes are related to innate immunity, apoptosis, extracellular matrix etc.

### Tandem 3′ UTR switching in host-pathogen interactions

**Figure 5:**
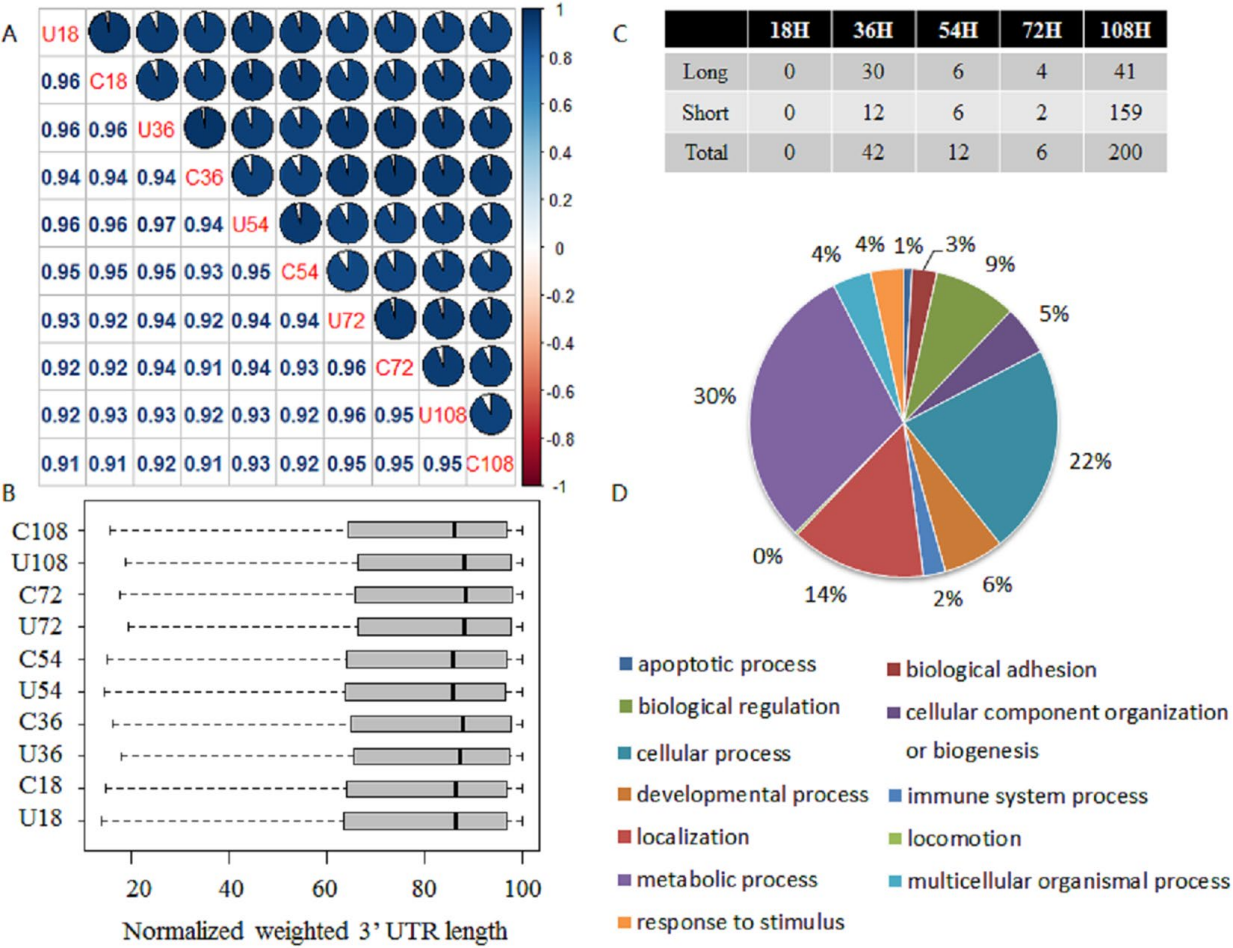
Tandem APA sites switching genes in host-pathogen interactions. (**A**) Pearson correlation coefficients of normalized weighted 3′ UTR length among samples. (**B**) Boxplot of normalized weighted 3′ UTR length among samples. (**C**) Summary of genes with tandem APA switching between samples at different time points. (**D**) Functional classification of tandem APA switching genes.

We next used our sequencing data to study the dynamic changes in overall 3′ UTR lengths and the regulation of APA after infection of MDV. We defined the 3′ UTR length of a gene as the weighted average length of all 3′ UTRs in the 3′ -most exon in the mRNA of the gene. In total, global profiles of 3′ UTR length of tandem genes were generally better correlated between samples than expression profiles, with Pearson correlation coefficients ranging from 0.91 to 0.97 (Fig. 5A). From the view of normalized average 3′ UTR length change between control samples and challenged samples at different time points, the infection of Md5 could result in decrease in average 3′ UTR length at 108 h (Fig. 5B).

We performed test of linear trend alternative to independence to compare the tandem 3′ UTR lengths of the unchallenged and challenged libraries at different time points^7, 17^. Genes with tandem 3′ UTR lengths that showed significant differences between libraries were defined as APA switching genes (Fig. 5C). The most of APA switching genes between samples were found at 36 h (42 genes) and 108 h (200 genes). In total, there were 243 genes (FDR < 0.01, Rcut < 0.1) with APA switching after infection of MDV, among which 169 genes tended to use shorter 3′ UTR, 66 genes tended to use longer 3′ UTR and 8 genes tended to use shorter or longer 3′ UTR at different time points. Six genes were selected to validate the APA sites switching with qRT-PCR (see Supplementary Fig. S2).

Through comparison of gene lists between APA switching genes and differentially expressed genes, 42 genes were found to under the regulation of two mechanisms simultaneously. Most of genes were under only one of the regulation mechanisms. Gene functional classification analysis by PANTHER^18^ showed that there was not too much difference in gene function between two lists of genes (Fig. 4C,D). These results showed that these two mechanisms cooperated and complemented in the host’s response to MD5 infection.

### APA switching between coding region and 3′ UTR

APA sites are located mainly in 3′ UTR and partly in coding region. APA sites in coding regions traditionally generate mRNA isoforms with premature translation termination, which could change amino acid sequences and influence common gene functions, while APA sites in 3′ UTR don’t. So we also tried to detect switching events between coding region and 3′ UTR.

**Figure 6:**
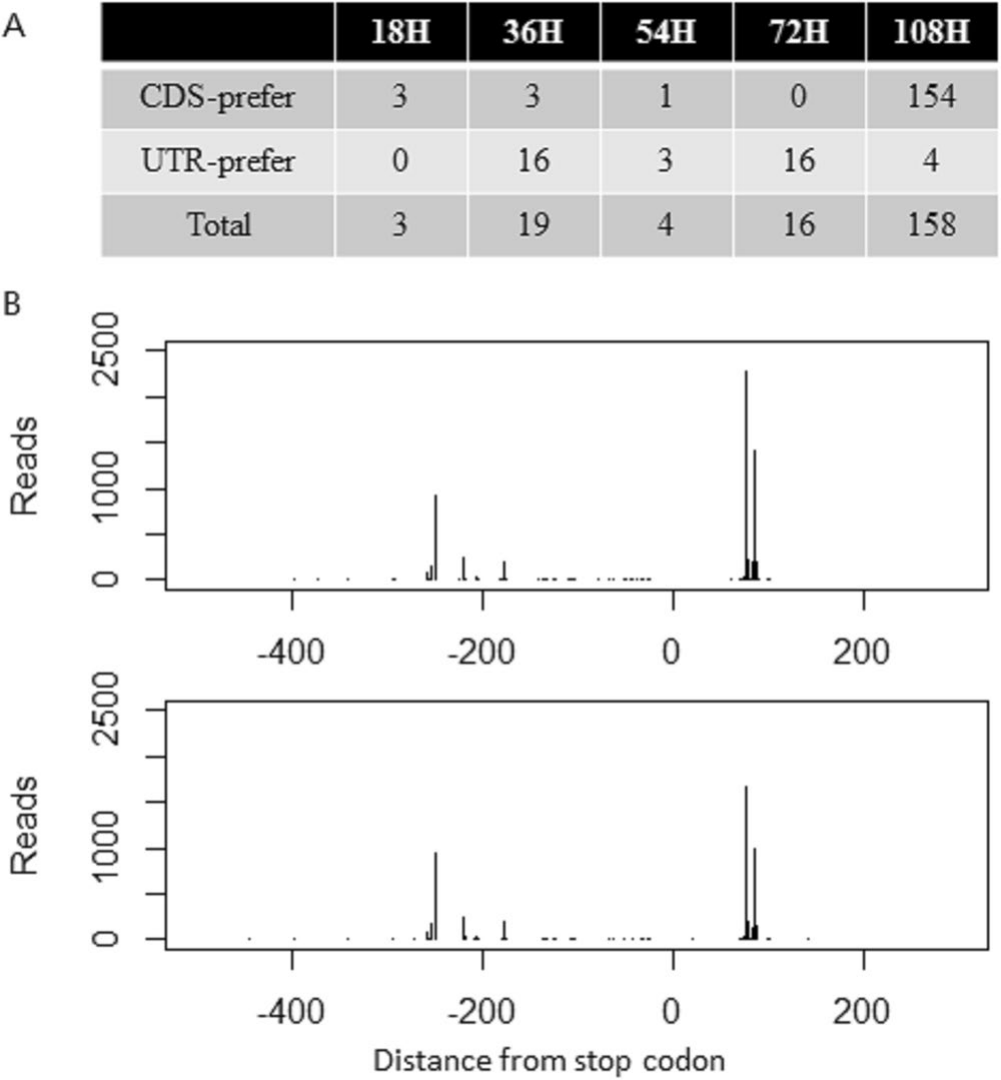
Genes with APA switching among coding region and 3′ UTR region. (**A**) Summary of genes with APA switching among coding region and 3′ UTR region between samples at different time points. (**B**) ATF4 occurred APA switching among coding region and 3′ UTR region at 18 h.

For APA sites in a gene, we classified these sites into two groups according to their location, namely coding region and 3′ UTR. Then we introduced chisq-squared test to evaluate whether existed significant switching between these two groups. If the usage of coding region APA sites improved, we called it CDS-prefer and otherwise UTR-prefer (Fig. 6). In total, 194 genes (FDR < 0.01) occurred switching events between coding region and 3′ UTR. At the early stage (18 h), we detected 3 genes preferred to use more APA sites within coding region including transcription factor ATF4, while no genes preferred to use more APA sites within 3′ UTR. At the late stage (108 h), we found more genes preferred to use more APA sites within coding region rather than within 3′ UTR. At the middle stage, it seemed opposite and more genes preferred to use APA sites within 3′ UTR.

### Cis-regulatory elements in APA switching genes

The APA switching of genes may be introduced by multiple reasons including 3′ end processing factors, transcription factors, RNA binding proteins, etc^2^. These factors must cooperate with cis-regulatory elements within genes to function. So we could analyze the 3′ UTR sequences of APA switching genes to figure out related cis-regulatory elements and even potential trans-factors.

To find out the potential cis-regulatory elements that could impact the usage preference of poly(A) sites, we first separated APA switching genes into different categories as before^17^. Then we got the proximal or distal major sites responsible for the most up-regulated and down-regulated poly(A) sites in “up-down” and “down-up” categories and used the upstream 150 bp 3′ UTR sequence to conduct 4–9 bp motif enrichment analysis. For the proximal poly(A) sites, down-regulated poly(A) sites tended to have more AU-rich elements on the upstream and more G-rich elements on the downstream, while up-regulated poly(A) sites tended to have more A-rich or AG-rich elements on the upstream and A-rich or U-rich elements on the downstream (Table 2). And for the distal poly(A) sites, down-regulated poly(A) sites tended to have more AU-rich elements on the upstream and downstream, while up-regulated poly(A) sites tended to have more C-rich elements on the upstream and downstream (Table 2). As a result, AU-rich elements showed significant correlations with the switching of poly(A) sites.

**Table 2:** Summary of cis-regulatory elements around the upstream and downstream of poly(A) sites in APA switching genes.

	Proximal sites		Distal sites	
	Up	Down	Up	Down
Upstream	AAAA, AGAG, AAAAA, AAAAAA	TATT, ATAT, AATA, TAAT, TATA, ATTT, TTAT	CTCC, CCCC, CAGC, CCTC, TCCC, TCCT, CCCCC	TATA, TTTT, ATTT, ATAT, TACA, TTAA, TTTA, TAAT, GTAT, TATT, ATTTT, TATTT, TTTTTT
Downstream	AAAA, TACA, TTTT, TGTA, AAAAA, TTTTTT, AAAAAA	TGGG, GGGG, GGGGG	GCGC, CCTC, CGCC, GCTC, CCCC, CGGG, GCTCC	TGTA, TTTT, AATG, AAAT, TAAA, ATGT, ATAAA, TTTTT

## Discussion

mRNA isoforms with different 3′ UTRs produced by APA are distinct in their stability, translation efficiency, and cellular location^3–6^. A comprehensive genome-wide assessment of APA genes is required for understanding APA involvement in biological processes. However, traditional quantification methods, such as quantitative PCR and microarray, cannot meet these requirements. Several recently developed strategies based on next-generation sequencing technology have revealed a large number of previously unidentified poly(A) sites. For example, 69.3% of poly(A) sites identified by the SAPAS strategy in human breast cancer cell lines were novel, 56.1% of DRS reads overlapped with previously annotated poly(A) sites. In the present study, fewer than a quarter of the identified poly(A) sites in the 3′ UTR of chicken genes were annotated in Ensembl annonations^19^. These newly identified poly(A) sites may dramatically change the analysis of APA and provide a more comprehensive and authentic profile of 3′ UTRs. Such profiles may ultimately help to explain how variation in 3′ UTRs is regulated in a given biological process, such as virus infection.

Given the complexities and limitations of MDV infection model system, we had two problems to deal with. First, there are no cell lines for MDV propagation, and the virus is generally grown in primary or secondary CEF^20^. Therefore, it is not possible to synchronize infections to the degree that would be easily achieved with other viruses. We chose different time points at 18 h, 36 h, 54 h, 72 h and 108 h p.i for sample collection, as these time points represent a relatively whole process in infection process from initial infection to visible plaques. Second, MDV is a cell-associated virus and therefore cannot be used at high multiplicities of infection^21^, resulting a partial infection of the cultured cells. This means that a large portion of the mRNA being purified at the time of harvest is from uninfected cells, which probably obscures the true magnitude of any differences observed. One must keep in mind that the differences observed here are from the result of mRNA levels containing both infected cells and uninfected cells.

By simultaneously measuring the expression and position of poly(A) sites, the SAPAS strategy provides a powerful and reliable method for this kind of transcriptome profiling^7^. Using this method, we present the genome-wide profiling of APA sites in host and pathogen. For genes with switched APA sites identified in our study, we did not observe a clear correlation between the length of the 3′ UTR and gene expression level. From an overall perspective, we observed a delay in time between the regulation of expression profiles and that of APA switching, which reflected the difference of these two mechanisms in the manner of regulation. Besides, 36 h and 108 h were two of the most regulated time points for gene expression level and APA switching events, while 54 h and 72 h were likely at a bottom. Considering the situation that the proportion of sequenced virus reads increased with time, we inferred that 54 h and 72 h referred to a transition state that early infected cells died and the number of new infected cells improved.

Through 3′ end enriched RNA-seq, we profiled the gene expression and usage of APA sites of both host and pathogen simultaneously. In total, our work focused on the dynamic gene regulation during Marek’s disease virus infection, including expression level and APA switching events. Our results gave a relatively comprehensive insight into dynamic host-pathogen interactions during Marek’s disease virus infection and contributed to the chicken genome annotation.

## Methods

### Cells culture and MDV infection

To identify cellular responses with MDV infection, chicken embryo fibroblast (CEF) cultures were prepared from ten-day-old specific-pathogen-free (SPF) chicken embryos. Secondary cultures were plated at a density of 1 × 10^7^ cells/flask and then infected by MD5 strain (5 × 10^4^ PFU/flask). The mock infection was used as control. Cells were collected at 18 h, 36 h, 54 h, 72 h, 108 h post-infection. In total, ten samples were used for library preparation. The Institutional Animal Care and Use Committee of Sun Yat-sen University, PRC, approved all the experimental protocols concerning the handling of chicken embryos. And all experiments were performed in accordance with the relevant guidelines and regulations.

### IVT-SAPAS library preparation and sequencing

The IVT-SAPAS libraries were prepared as described previously^7, 22^. Briefly, total RNA was extracted from samples using TRIzol (Invitrogen) according to manufacturer’s instruction. Approximately 2 μg total RNA was randomly fragmented by heating. First-strand cDNA was synthesized by reverse transcription with an anchored oligo d(T) primer and a 5′ template switching adaptor. PCR was then performed to amplify the cDNA, and the number of cycles was optimized to ensure that the double-strand cDNA remained in the exponential phase of amplification. Then fragments of 250–500 bp in size were excised and purified by Agencourt Ampure magnetic bead (Beckman Coulter) according to manufacturer’s instruction. The average size was determined by Agilent 2100 bioanalyzer (Agilent Genomics). The final pooled fragments were quantified and sequenced from the 3′ end with Illumina GA IIx.

### Profiling analysis of alternative polyadenylation (APA) sites

The SAPAS-generated raw reads were filtered and trimmed for quality control utilizing Perl script, and then mapped to the chicken genome galGal4 assembly^23^ and Marek’s disease virus genome MD5^24^ respectively by applying Bowtie^25^. After internal priming filtering, the resulting uniquely mapped reads were clustered to define poly(A) sites as described previously^7^. The genomic location of poly(A) sites were defined according to gene annotations. The poly(A) signal of each site was selected based on its genomic sequences.

### Alternative polyadenylation (APA) sites switching analysis

Here we consider two types of APA switching, namely tandem APA switching and switching between coding region and 3′ UTR. The total read counts from each sample were normalized to one million reads, and poly(A) sites with two or more normalized reads were used for further analysis. A combined model to test tandem APA switching events was performed as described before^7, 17^. Briefly, genes with significant APA switching between the infected and normal cells were identified by the linear trend test and the independence test. As to switching between coding region and 3′ UTR, we classified APA sites in a gene into two groups according to their location, namely coding region and 3′ UTR. Then we introduced chisq-squared test to evaluate whether existed significant switching between these two groups. If the usage of coding region APA sites improved, we called it CDS-prefer and otherwise UTR-prefer.

### qRT-PCR validation of gene expression

Briefly, the total RNAs were isolated with the TRIzol (Invitrogen) and purified with an RNeasy mini Kit (QIAGENE), reverse-transcribed into cDNA using the PrimeScript RT reagent kit (Takara), and amplified using a LightCycler 480 real-time PCR machine (Roche).

### qRT-PCR validation of APA switching genes

Genes with significant length differences in their 3′ UTRs between libraries were chosen for quantitative real-time RT-PCR (qRT-PCR). The poly(A) sites of these genes were divided into two supersites (the proximal and distal sites), and the region upstream of the supersites was targeted for qRT-PCR. Double-stranded cDNA was synthesized from total RNA using the PrimeScript RT reagent kit (Takara). qRT-PCR was performed on a LightCycler 480 real-time PCR machine (Roche) using the SYBR Pre- mix Ex Taq II kit (Takara) in a 10 ml reaction system. All samples were analyzed in triplicate.

### Cis-regulatory elements analysis

To obtain the usage ratio of each poly(A) site in a gene, the supporting reads of each poly(A) site were divided by the sum of the supporting reads of all poly(A) sites in a gene. Then the change of usage ratio change was defined as the difference between two samples. Thus, a negative number means a change “down” and a positive number means a change “up”. The absolute value reflects the amplitude of the ratio changing. A bigger absolute value indicates an increased poly(A) site usage switching. Neighboring sites with same “up” or “down” trend are considered as single sites, and those whose absolute value are close to zero can be merged to neighboring sites, according to the situation^17^. Kmer motif enrichment analysis from 4 bp to 9 bp was performed on the upstream 150 bp of the 3′ UTR sequence. After normalization, binomial test was used to calculate the significance of motif enrichment in R.

### Data availability

The datasets generated during and/or analysed during the current study are available in the Gene Expression Omnibus with the primary accession code GSE92567.

## Electronic supplementary material

Supplementary information
