# Supplementary material for: A novel method for genome-wide profiling of dynamic host-pathogen interactions using 3′ end enriched RNA-seq
Source: Sci Rep. 2017 Aug 17;7:8681. doi: 10.1038/s41598-017-08700-9 (PMC5561256; doi:10.1038/s41598-017-08700-9)
Supplement: Supplementary file 1 — Supplementary information [file 41598_2017_8700_MOESM1_ESM.pdf]

A novel method for genome-wide profiling of dynamic host-pathogen interactions using 3’ end enriched RNA-seq

Jie Li[1,*], Liangliang He[1,*], Yun Zhang[1], Chunyi Xue[1], Yongchang Cao[1]

Supplement Figure S1. qPCR validation of the gene with expression change. For each gene, the expression level change was validated at different time points. The fold changes generated through RNA-seq are shown in red lines, and those qPCR results are shown in blue lines.

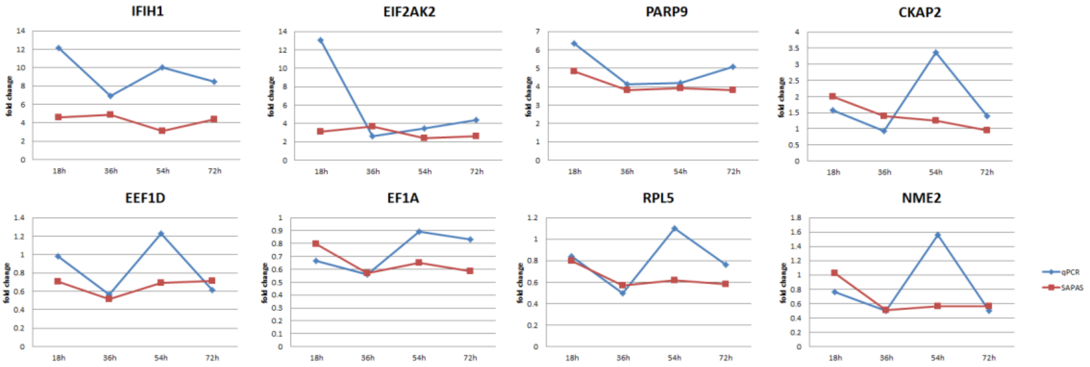

Supplement Figure S2. qPCR validation of the gene with tandem APA switching. The poly(A) sites of each gene were divided into two supersites (the proximal and distal sites), and the region upstream of the supersites was targeted for qRT-PCR. The APA sites usage of distal sites was calculated as the ratio between the expression of distal sites and the total gene expression. The relative change of APA sites usage was got as the ratio between the APA sites usage of distal sites after the infection and that before infection. If this value is less than 1, it means after infection the gene promotes to use proximal APA sites. Otherwise, it means after infection the gene promotes to use distal APA sites.

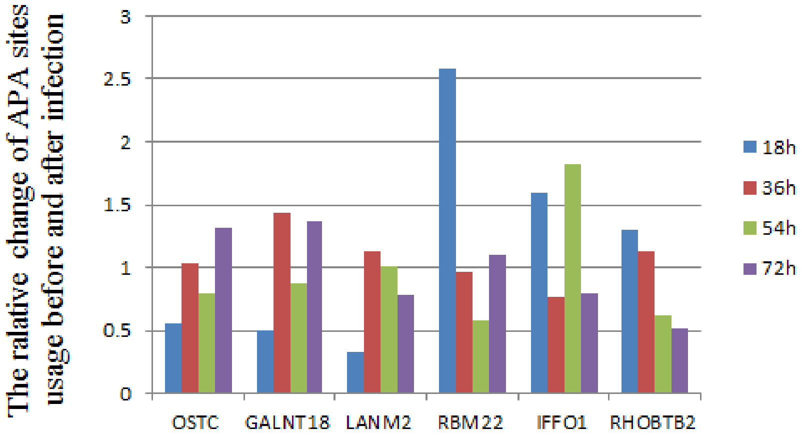

Supplement Table S1. List of genes with significant expression changes. Genes that changed more than 2-fold and had a false discovery rate (FDR) less than 0.01 were considered to be genes regulated differentially.

LY6E	MT4	TGFBR3	CCDC3	UBR7	GAL10	CDH11	HGF/SF	CRCP	ATL1
CST3	IFI35	CADM1	GLG1	FBXO9	BRT-1	ALDH18A1	DNTTIP1	[4Fe-4S]	PDLIM7
THBS1	ELN	SGK1	KANK1	Lpin1	RPL8	GSTA3	E2F4	PRPSAP1	GNAS
CSRP2	LMF2	COPB1	NDST1	PTK7	FBLN1	DAP	NT5C3B	FGFR1OP2	RPL4
ISG12(2)	RSU1	EMP2	NHSL2	DEPDC6	NREP	SLCO3A1	CNDP2	DEF8	ALDOC
PDLIM1	PLD1	RGS3	PPAPDC2	C12ORF57	WBSCR27	HMOX1	ITGB3	PTGES2	POLDIP2
MYH10	UBP1	MON1A	ULK3	AS3MT	GEM	SLC35F6	col14A1	LSM1	EIF4E
ADM	CD44	C10ORF58	LAMTOR2	TNKS1BP1	DTX3L	SLC9A3R1	RASSF2	HDHD3	REEP5
ACTG2	CTNNA1	EGLN1	RASA3	FBXW5	DKK2	SESTD1	AP2B1	FAM214A	LUM
TAGLN	CDK2AP1	PLBD2	SLC7A11	ARPC4	HHIP	MARCKSL1	FAP	GLI2	CTSK
COL9A2	CLIC2	SAMHD1	NIP7	AKAP1	FAM107B	GM2A	OCRL	IFFO1	IARS
Ex-FABP	HPGDS	PRRC1	OSTN	LIMD2	OTUD4	CASP3	gadd45	AKR1D1	PDIA3
LDHA	RAD21	RHOBTB2	WDFY3	BCAR1	APOA1	JMJD8	SNX12	NRBP1	OCIAD1
AKAP12	SPP1	UBE2E3	METTL13	MYO18A	FMOD	TMBIM1	LMAN2	GCC1	EIF3E
C1S	CX3CL1	PSMG1	SMIM5	CEP68	TMEM115	TRAIP	SPTBN1	YAF2	PTGFRN
RRM2	OAT	IGFBP7	CASP9	TAF12	KLF4	PENK	SH3GLB1	TLK1	ZNF609
MYL9	EIF5B	TNC	DPM2	TMEM214	NDRG1	ADAM9	MPST	NPB	TUBB4B
TPN	MEIS2	SFXN1	STOML1	FARSB	RPL10A	MVD	IL11Ra	RBP5	CTSL2
TNFIP6	OGN	ERBB2IP	ZNF207	PPIB	C1QB	METRNL	ACKR4	TMEM138	MORN4
EF1A	TTC8	PHGDH	NPLOC4	AKAP7	SLIT2	CHAC1	NOV	PHB	SFRP2
MGP	PAM	MKX	MIB1	CDKN2B	CKAP2	PHF11	XPR1	PC7	GLO1
CMPK2	TRANK1	RPL24	AVPR2	SRSF7	PKP2	PODN	Ii	HIF1AN	RPL19
FHL5	CSRP1	NCAPD2	EEPD1	NECAP1	ADAR	CDH17	PLEKHH3	5-Mar	FAM177A1
DHX15	RPLP0	DNAJB6	PCMT1	KIAA0355	HTRA1	MXRA7	SYBU	BDH1	EN1
SQSTM1	SCP2	CASP7	TGS1	PSMD3	TBRG4	OCC-1	EMP1	ZNF592	NME2
RPL37A	KDELR2	Sulfl	FTSJ3	B4GALT6	RSFR	EIF2S3	SCAP	MCFD2	C
SLC25A6	PPAP2B	IQCJ-SCHIP1	SEPHS1	CHTOP	NOC4L	NFKBIZ	DMB2	TMED3	
angiopoietin-2	FAM20A	NPR2	CNTF	USP5	GDPD5	CENPH	IL34	RIL	
CFL2	PSAT1	SQLE	NPNT	RPL22L1	PARP9	IK	ZNF335	SGK3	
JAC	ADAMTS2	BET1	ALCAM	MORF4L1	PCNP	MUSTN1	TYSND1	FAN1	
ITPR3	CYBRD1	5-Sep	TMEM120A	TBC1D12	MYH9	SPR	PITHD1	TAF7	
CDCA7	DPT	SEC31A	SIDT2	USP12P1	CKS1B	SDC1	RGS14	RPLP2	
IL1RL1	CITED4	ASPN	HGS	FNDC1	RAB34	CDKN1A	SERINC2	LIMK1	
RPS3A	FKBP7	SUMO2	ATG9A	MRPS6	WIPI1	SERPINI1	BCL2L1	SLC25A39	
LGALS3	SLC29A1	CSTB	RRP12	PEF1	S100A4	CDC42EP4	THY1	PPP3CA	
CCK	TEX264	RNF19B	GPD1L	ANXA1	EIF2AK2	CLCN7	FLAD1	FBXO42	
ANG-1	CCLI10	C1QTNF1	TWF2	C1QTNF5	RARRES1	XBP1	SLC12A9	SLC19A1	
KRT14	COPG1	BLOC1S5	SLC52A3	STAG2	ARHGEF4	RPL3	UPP1	ZNF217	
CHAF1B	TMEM53	UBE2D1	PTPN9	OSBPL11	ETV4	NPM3	TBC1D22B	NOVA1	

BMPER	ADAMTS1	RSPO2	TRMU	AP3M2	SNX10	VPS11	PHLDB2	MCAT	
IFI27L2	TGFB3	GPC1	SDF2	RAP1A	CO6	GARS	WDR24	HYAL1	
Enpp2	YIPF1	FGD3	CYB5RL	TCIRG1	FERMT2	SSR2	LRP8	UBE4A	
EFEMP1	CTPS1	EPT1	TUFT1	SIGMAR1	CAPN1	DDHD1	CD1.2	STUB1	
KIF26A	PPP2R4	MRPL10	TDP2	AGAP3	NCKAP1	DHX36	DYNLL1	GTF3C2	
NFKBIA	CPD	RFTN1	ANKRD10	TMEM222	PYURF	RPS6	SYDE2	GIGYF2	
MAOB	BTG1	SHC4	CHPF2	NAA38	MCL1	ZFP91	C2orf40	PSMC5	
MYLK	IRF-3	TRAFD1	E2F1	PHPT1	IFIH1	SFSWAP	ADIPOR1	SPON2	
BFIV21	SGSH	SPTSSA	C21H1ORF144	BCL9	HIC1	TRAPPC8	PSPH	MED21	
TLCD1	CLEC3B	SIKE1	RARRES2	SPPL2A	NMI	AP3S2	PFKL	MMP15	
EEF1D	ERAP1	GPT2	PPPDE1	FADS1	C14orf79	QDPR	SLC37A2	EIF2B4	

Supplement Table S2. List of genes with significant tandem APA switching. A false discovery rate (FDR) less than 0.01 were considered to be genes regulated differentially.

KLF3	TMCO1	GTF2H1	FNDC3B	ANXA5	RNF4	SLCO3A1	COX19	DPYSL3	INTS6
RASSF2	VAT1	PFKFB3	SFRP2	RANGAP1	GABPA	FAM63B	OCC-1	ZC3H6	ARL6IP5
MAN2A1	AGFG1	FBXO45	SP3	RPL7L1	SYNCRIP	PSMC3	TCF25	ITPR1	CDCP1
PSMF1	PINK1	SNRPD3	SEC62	ST13	OSR2	RNF13	CSRP1	TWSG1	ER81
CSRP1	SCPEP1	UPF2	SIPA1L1	ACTR3	NOV	GTF3C5	RHOBTB2	CRNKL1	C3H2ORF43
PPP6C	Mlx	CHKA	SLC39A9	FKBP9	NDRG1	WDR43	TUFT1	RIN2	PPP4R2
SNAP29	C21H1ORF144	FADS1	RARRES1	KBTBD2	CASK	PYGB	GAPVD1	TTL	OAT
RAB14	AXIN2	ITGB1	PALLD	JOSD1	PXDN	SIKE1	RSPRY1	DSTN	COG5
RAP1A	TM2D3	VIMP	MRPS18A	ACVR1	col14A1	RBPMS2	SSU72	MARK1	EIF4G2
GIGYF2	DNAJB12	SUDS3	DHX36	SDHA	TRAM2	RER1	PPP2R4	C4orf27	SPON1
9-Sep	RBM22	YIPF5	LTBP1	CLPTM1L	CYFIP1	ZIC1	INPP5E	OSTC	SMO
FAM65A	BRAP	ARCN1	RNF11	TBL1X	EIF5B	Gga.5519	CYP27A1	GALNT7	GALNT18
SDF4	AP4E1	YME1L1	EPS15	SRSF7	DNAJC3	THY1	POLDIP2	SERPINH1	H3F3B
HEXA	CERCAM	USP12P1	YIPF4	GPR155	INTS6	PMP22	KPNA6	HIF1A	SAR1B
NCOA4	DHX30	ADCY9	ZFYVE9	TMEM33	UBL3	H3F3B	DDX5	ANXA5	PRKCDBP
CHMP4B	DKC1	HN1	PODN	MRPL51	MED17	PRKCDBP	TM2D3	KIAA1468	ARMT1
HSD17B4	FAM96B	TRIM8	PPPDE1	SEPHS1	TMEM135	LOX	TOR1A	CHMP5	LMAN2
CTNNA1	USP32	SEC13	EXOC8	SGTB	CKI	N-RAS	CPD	UBQLN4	CBLL1
CDC73	NOLC1	CRNKL1	PPP2R5C	NDC80	PSMD3	USP47	TIMM8A	SEC63	RAB12
MAT1A	IPO7	AP2M1	DYNC1LI1	OSBPL1A	RWDD1	METRNL	ZNF598	TRAM1	PDCD10
PAIP2	IL11Ra	STAG2	KIF26A	B4GALT6	TGFB2	WBP1L	SNX12	OSR2	EIF1
HSD17B7	ADAMTS2	WASL	DDX47	PCNP	WDR44	CARHSP1	RAB7A	SNX17	GNG12
DNAJC9	HNRNPA0	ISM1	HEBP1	TGS1	PDCD7	SRP9	LUC7L	TM9SF2	OGN
UNC119B	C12orf66	IMPA1	CTNNBIP1						

Supplement Table S3. List of genes with significant APA switching between coding region and 3' UTR. If the usage of coding region APA sites improved, we called it

CDS-prefer and otherwise UTR-prefer. A false discovery rate (FDR) less than 0.01 were considered to be genes regulated differentially.

PHLDB2	DDHD1	ADD1	CNOT6	PPM1D	NDRG1	NUP153	SH3KBP1	SET	MYH10
LUC7L3	FKBP9	TRANK1	YBX1	UPF2	C19H17ORF85	YWHAH	IQGAP1	SYT11	ITGB1BP3
AFAP1	KTN1	ATF4	COL4A1	SMARCA2	OSBPL5	USP7	NCKAP1	AP1M1	NF2
SRSF11	avenaII	ACBD3	TM2D1	CDK2AP1	C1orf198	CCDC25	DEK	PPM1B	PITPNB
ACTG2	MTDH	ZNF207	ZFC3H1	ANO5	DST	TCERG1	ZNF326	CCL4	ADAM9
SRRM1	FNDC1	HDAC2	LETM1	ADAMTS2	ADD3	MIB1	EHD3	HMGCS1	C20H20ORF43
ENSGALG00000013365.4	ENSGALG00000015805.3	NOC3L	ENSGALG00000003235.4	ZC3H13	SFPQ	RALBP1	ENSGALG00000020557.3	ENSGALG00000014190.4	TOP2B
CDC42BPB	EXOC3	WNK1	SRSF4	PAXBP1	RPLP2	RPL27	TAF3	DDX5	ANKRD11
BOD1L1	USP12P1	IFT52	IREB2	SDF4	FAM21A	HNRNPDL	YAF2	RPLP1	MRPS6
YWHAE	UHRF1	FASN	STRN3	MYO9B	TOP1	PABPC1	RAP1B	RNF20	THRAP3
FAM107B	FAM177A1	PUM2	ECM2	NARS	ASUN	ALDH18A1	ARHGAP21	fn1	PNRC1
ATRNL1	RPAP1	PPP4R1	URI1	CAMSAP2	RSRC2	RAB3GAP1	MATR3	LMAN1	EIF5B
COL5A2	PPP1R8	LRRC16A	ZEB1	ABCC1	ROCK1	COPB1	CSNK1D	OSF-2	EEF1B2
DDRGK1	RASA3	DPYD	TMX4	EGFL6	CKAP4	SHISA5	PRKCDBP	AKAP12	HSD17B12
XPA	SLC16A3	SBNO1	SREK1	BMPER	PDE3B	RANBP2	MDN1	SPTBN1	EIF5
DCN	TAX1BP1	SACS	FNDC3B	ATP6V1E1	GLI2	RBM25	PRPF6	IK	FAM135A
HECTD1	REV3L	WDFY3	RAPGEF2	CCPG1	RIF1	SPRY1	MYLK	UBE2H	MORF4L1
7-Sep	ANP32E	FBLN1	RIN2	HN1	ABCE1	vimentin	UTRN	LSP1	PLEKHA5
USP25	TBC1D12	EIF3A	BIRC6	POC1B	CEP170	AKAP1	NKAP	RPL5	APLP2
HSPA5	PHF21A	IPO7	SVIL						
